# Development and validation of a preoperative nomogram for predicting contralateral occult carcinoma and central lymph node metastasis in T1b-T2 stage papillary thyroid carcinoma

**DOI:** 10.3389/fendo.2026.1891976

**Published:** 2026-08-19

**Authors:** Yu Zhao, Yaqi Zhao, Runze Li, Yuming Luo, Silin Xu, Yalan Zeng, Chong Lu, Chunping Liu

**Affiliations:** 1 Department of Breast and Thyroid Surgery, Wuhan Union Hospital, Tongji Medical College, Huazhong University of Science and Technology, Wuhan, China; 2 Department of Breast and Thyroid Surgery, The Second Affiliated Hospital of Hainan Medical University, Haikou, China

**Keywords:** central lymph node metastasis, nomogram, occult carcinoma, papillary thyroid carcinoma, predic risk

## Abstract

**Background:**

Thyroid cancer is one of the fastest-growing malignancies worldwide, with papillary thyroid carcinoma (PTC) accounting for approximately 85-90% of all cases. For patients with T1b-T2 PTC, the choice between thyroid lobectomy and total thyroidectomy remains controversial. Postoperative pathological examination reveals contralateral occult carcinoma in 20-40% of patients, while contralateral central lymph node metastasis (CLNM) represents a key prognostic factor. However, reliable and practical preoperative tools to support individualized surgical decision-making are currently lacking. This study aimed to develop and validate a preoperative prediction model for assessing the risk of contralateral occult carcinoma (OTC) and contralateral CLNM in patients with T1b-T2 PTC, thereby facilitating individualized surgical management.

**Methods:**

This retrospective study enrolled 932 patients with T1b-T2 PTC who presented with unilateral nodules on preoperative ultrasound and underwent total thyroidectomy with bilateral central lymph node dissection. All 932 patients were included in the contralateral OTC prediction model, and 730 patients were included in the contralateral CLNM prediction model. Nine machine learning algorithms and logistic regression models were trained and compared using five-fold cross-validation. Key predictive features were selected through multivariate logistic regression and random forest. Model performance was evaluated using the area under the receiver operating characteristic curve (AUC), calibration curves, the Hosmer Lemeshow test, and decision curve analysis.

**Results:**

Feature selection identified four independent predictors of contralateral OTC: malignant isthmic nodule, sum of the longest diameter (SLD), number of malignant nodules, and capsular disruption. Five predictors were identified for contralateral CLNM: isthmus adjacent nodule, sex, SLD, age, and chronic lymphocytic thyroiditis (CLT), with CLT showing a negative association with CLNM.

Logistic regression model demonstrated the best performance for OTC prediction (AUC = 82.00%). For contralateral CLNM, logistic regression achieved an AUC of 79.00%, comparable to that of the best-performing machine learning model (glmBoost, AUC = 79.10%), with no statistically significant difference observed between the two models. Model calibration was satisfactory, as demonstrated by calibration curves and the Hosmer–Lemeshow test (P = 0.5097). Decision curve analysis further indicated favorable clinical net benefit.

**Conclusion:**

This study developed a logistic regression-based preoperative predictive tool to assess the individualized risk of contralateral occult carcinoma and contralateral CLNM in patients with T1b-T2 PTC. To our knowledge, this is the first dual-outcome prediction model specifically designed for this patient population. The prediction results were visualized using two nomograms and two web-based applications, which may assist clinicians in making individualized surgical decisions before surgery. Specifically, low-risk patients may be considered for thyroid lobectomy to avoid overtreatment, whereas high risk-patients may undergo total thyroidectomy to reduce the risk of recurrence.

## 1 Introduction

Thyroid cancer ranks among the most rapidly increasing malignancies globally in terms of incidence, with PTC accounting for approximately 85%-90% of all thyroid cancer cases (1, 2). Despite PTC’s generally favorable prognosis, its incidence has continued to rise worldwide over recent decades. Notably, China has reported an annual increase of up to 20.1%, representing the fastest growth rate among all malignancies (3, 4). With the widespread adoption of high-resolution ultrasound and other diagnostic modalities, an increasing number of patients are being diagnosed at earlier stages, particularly those with T1b-T2 disease (tumor diameter > 1 cm and ≤ 4 cm), who now representing a significant proportion of cases (5).

For patients with T1b-T2 PTC, the optimal extent of surgery remains controversial, particularly regarding the choice between thyroid lobectomy and total thyroidectomy (6, 7). According to the American Thyroid Association (ATA) guidelines, thyroid lobectomy may be considered as an initial treatment for patients without high risk features, as it can minimize the likelihood of surgery-related complications, such as permanent hypoparathyroidism and recurrent laryngeal nerve injury, while preserving residual thyroid function (8). However, before finalizing this surgical strategy, careful preoperative evaluation of vocal cord motility is essential, as it directly informs the risk of recurrent laryngeal nerve injury and helps tailor the operative plan (9). However, this strategy is challenged by the relatively high incidence of occult carcinoma in the contralateral lobe. In clinical practice, a considerable proportion of patients initially diagnosed with unilateral disease are subsequently found to have contralateral occult carcinoma on postoperative pathological examination (10, 11). Occult carcinoma refers to malignant lesions that are not detected by preoperative examinations such as ultrasound or fine-needle aspiration biopsy (FNAB), but are identified only after surgery (10–14). Previous studies have reported that the incidence of contralateral occult carcinoma in patients with PTC ranges from 20% to 40% (12, 15, 16). In such cases, patients who initially undergo lobectomy may require a second surgery, thereby increasing the risks of complications, psychological distress, and healthcare costs.

In addition to contralateral OTC, contralateral CLNM is another key factor influencing surgical planning. The central compartment is the most common site of lymph node metastasis in PTC, with reported incidences ranging from 30% to 80% (16, 17). Accumulating evidence has confirmed that lymph node metastasis is independently associated with locoregional recurrence and reduced disease-specific survival (18). For patients with unilateral PTC, contralateral CLNM cannot be adequately managed through lobectomy alone, potentially leading to incomplete lymph node clearance and an increased risk of recurrence. Therefore, accurate preoperative assessment of contralateral CLNM is essential for optimizing individualized surgical strategies.

Currently, preoperative assessment primarily relies on high-resolution ultrasound. However, the sensitivity of ultrasound in detecting CLNM remains unsatisfactory, especially for micrometastases or lymph nodes located posterior to the thyroid gland (19). Its ability to detect occult carcinoma is similarly inadequate. Although computed tomography (CT) and FNAB can provide additional information, their sensitivity and specificity remain inadequate for reliable preoperative risk stratification. Moreover, FNAB is an invasive procedure, limiting its routine use in clinical practice (20).

Therefore, how to accurately predict the presence of contralateral OTC and contralateral CLNM in patients with T1b-T2 stage PTC before surgery has become an important unmet clinical need. Most previous studies have focused either on identifying risk factors for a single outcome or on constructing separate prediction models. To date, an integrated preoperative prediction model simultaneously evaluating both contralateral OTC and contralateral CLNM in the T1b-T2 population remains lacking. In recent years, machine learning algorithms have shown considerable potential in medical prediction tasks by overcoming some of the limitations of traditional statistical methods (21, 22). Therefore, based on preoperative clinical data from patients with T1b-T2 stage PTC, including demographic characteristics, ultrasound features, and laboratory findings, this study constructed predictive models using machine learning algorithms to estimate the risks of contralateral OTC and contralateral CLNM before surgery. With these models, clinicians can perform individualized preoperative risk stratification. Specifically, low-risk patients may be suitable candidates for thyroid lobectomy to avoid overtreatment, whereas high-risk patients may benefit from total thyroidectomy combined with central lymph node dissection to reduce the risk of recurrence. This study aims to provide evidence-based support for individualized surgical decision-making in T1b-T2 stage PTC.

## 2 Methods

### 2.1 Patient and study design

This retrospective study collected clinical and pathological data of patients with PTC who underwent initial surgery at Union Hospital, Tongji Medical College, Huazhong University of Science and Technology in Wuhan, China, between May 2020 and May 2024.The inclusion criteria were as follows: (1) complete clinicopathological records; (2) preoperative ultrasound showing unilateral thyroid nodules, with postoperative clinicopathological confirmation of PTC; (3) treatment with total thyroidectomy with bilateral central lymph node dissection; (4) Primary tumors were classified as T1b–T2 stage with no evidence of distant metastasis. For patients with multiple malignant nodules, the sum of the longest diameters (SLD) was calculated as the arithmetic sum of the longest diameters of all pathologically confirmed malignant nodules; and (5) no prior history of other cancers or cervical radiation exposure.

**Figure 1:**
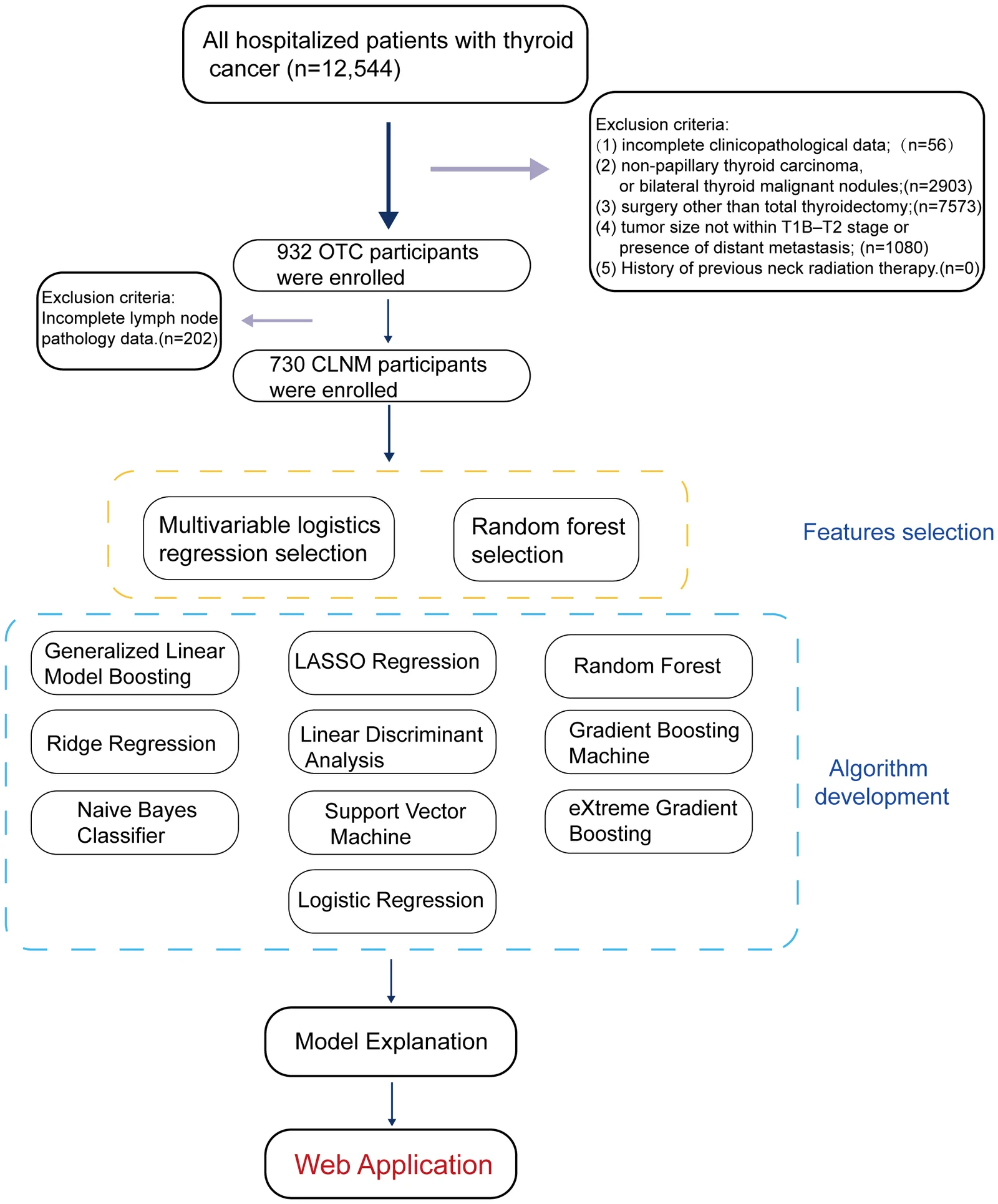
Flow chart of the study design.

Ultimately, 932 patients were included in the prediction model for contralateral OTC, whereas 730 patients were included in the model for contralateral CLNM (**Figure 1**).The study was conducted and reported in accordance with the Transparent Reporting of a Multivariable Prediction Model for Individual risk or Diagnosis (TRIPOD) statement. Given the exploratory nature of the study and the available sample size, the cohort was not randomly divided into separate training and validation sets. Instead, the entire cohort was used for model development, and internal validation was performed using bootstrap resampling.

### 2.2 Feature selection

The baseline characteristics of the enrolled patients are presented in **Table 1**. A total of 11 clinicopathological features were initially considered as candidate predictors. Other potentially important variables, such as BRAF V600E mutation status, family history of thyroid cancer, preoperative thyroglobulin levels, and ultrasound elastography features, were not included in the present study, as they were not routinely examined in clinical practice and had substantial missing data, which could have introduced bias if imputed or included. Feature selection was performed using an integrated strategy combining multivariate logistic regression analysis and the random forest algorithm, thereby leveraging the complementary strengths of machine learning approaches and traditional statistical methods.

**Table 1:** Patient demographics and baseline characteristics( contralateral OTC).

Characteristics	No (n=547)	Yes (n=385)	P value
BMI			
Mean ± SD	23.32±3.38	24.08±3.50	<0.001*
<24	502 (68.5%)	69 (59.0%)	
≥24 and<28	185 (25.2%)	35 (29.9%)	
≥28	46 (6.3%)	13 (11.1%)	
Age			0.181
≤55	473 (86.5%)	345 (89.6%)	
>55	74 (13.5%)	40 (10.4%)	
Mean ± SD	40.38±11.95	40.63±11.55	
Gender			0.096
Female	413 (75.5%)	271 (70.4%)	
male	134 (24.5%)	114 (29.6%)	
CLT			0.086
No	328 (60.0%)	253 (65.7%)	
Yes	219 (40.0%)	132 (34.3%)	
Number of malignant nodules reported by ultrasound			<0.001
1	631 (86.1%)	71 (60.7%)	
≥1	102 (13.9%)	46 (39.4%)	
SLD (cm)			
Mean ± SD	1.51±0.54	1.76±0.71	0.000*
Calcification reported by ultrasound			0.009
No	73 (13.3%)	77 (20.0%)	
Yes	474 (86.7%)	308 (80.0%)	
Anteroposterior-to-transverse ratio			0.804
<1	314 (57.4%)	225 (58.4%)	
≥1	233 (42.6%)	160 (41.6%)	
Malignant nodules located in the isthmus			<0.001
No	537 (98.2%)	335 (87.0%)	
Yes	10 (1.8%)	50 (13.0%)	
Malignant nodules located near the isthmus			0.14
No	515 (94.1%)	352 (91.4%)	
Yes	32 (5.9%)	33 (8.6%)	
Capsular disruption			0.006
No	110 (20.1%)	50 (13.0%)	
Yes	437 (79.9%)	335 (87.0%)	

OTC, contralateral occult carcinoma; SD, standard deviation; CLT, chronic lymphocytic thyroiditis; SLD, sum of the longest diameter. For multifocal lesions, SLD was calculated as the sum of the longest diameters of all malignant nodules; for unifocal lesions, SLD equaled the single tumor diameter; *indicates the p values were obtained from the Kruskal–Wallis test, and others were obtained from the Chi-square tests.

**Figure 2:**
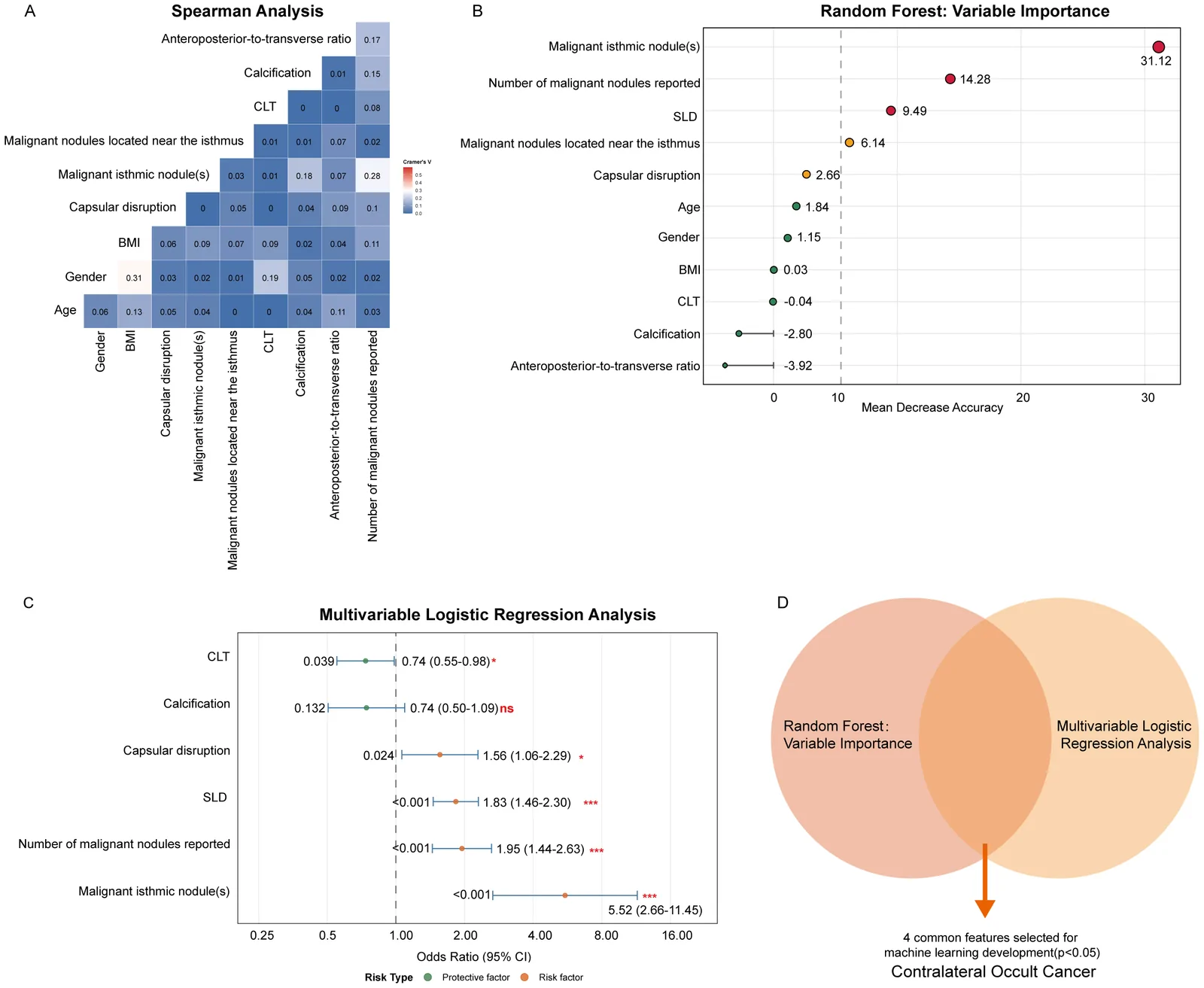
Comprehensive analysis of multiple variables to identify significant features. **(A)** The correlation heatmap between the variables. Multicollinearity analysis. spearman correlation coefficient and Cramer’s V correlation coefficient was performed to assess correlations among variables, and a heatmap was generated based on the correlation coefficients. **(B)** Random forest-based feature selection algorithm. Features marked in red and orange were identified as important features. **(C)** Forest plot of significant variables identified by multivariable regression analysis (P < 0.05). **(D)** Venn diagram illustrating the overlap of features selected by the random forest algorithm and multivariable logistic regression analysis.SLD, sum of the longest diameter; CLT, chronic lymphocytic thyroiditis; *p<0.05; **p<0.01; ***p<0.001; ns,not significant.

To minimize the influence of multicollinearity, Spearman correlation analysis (**Figure 2A**) and variance inflation factor (VIF) analysis were first conducted (**Supplementary Table 1**). Subsequently, the random forest algorithm was applied to identify variables with high predictive importance. Multivariate regression analysis was then performed to determine the odds ratios (ORs) and confidence intervals (CIs) for each predictor. An OR>1 indicated an increased risk of the outcome, whereas an OR<1 suggested a protective effect. By integrating the results of the above two methods, the final set of predictive features for model construction was determined.

### 2.3 Model construction and evaluation

**Figure 3:**
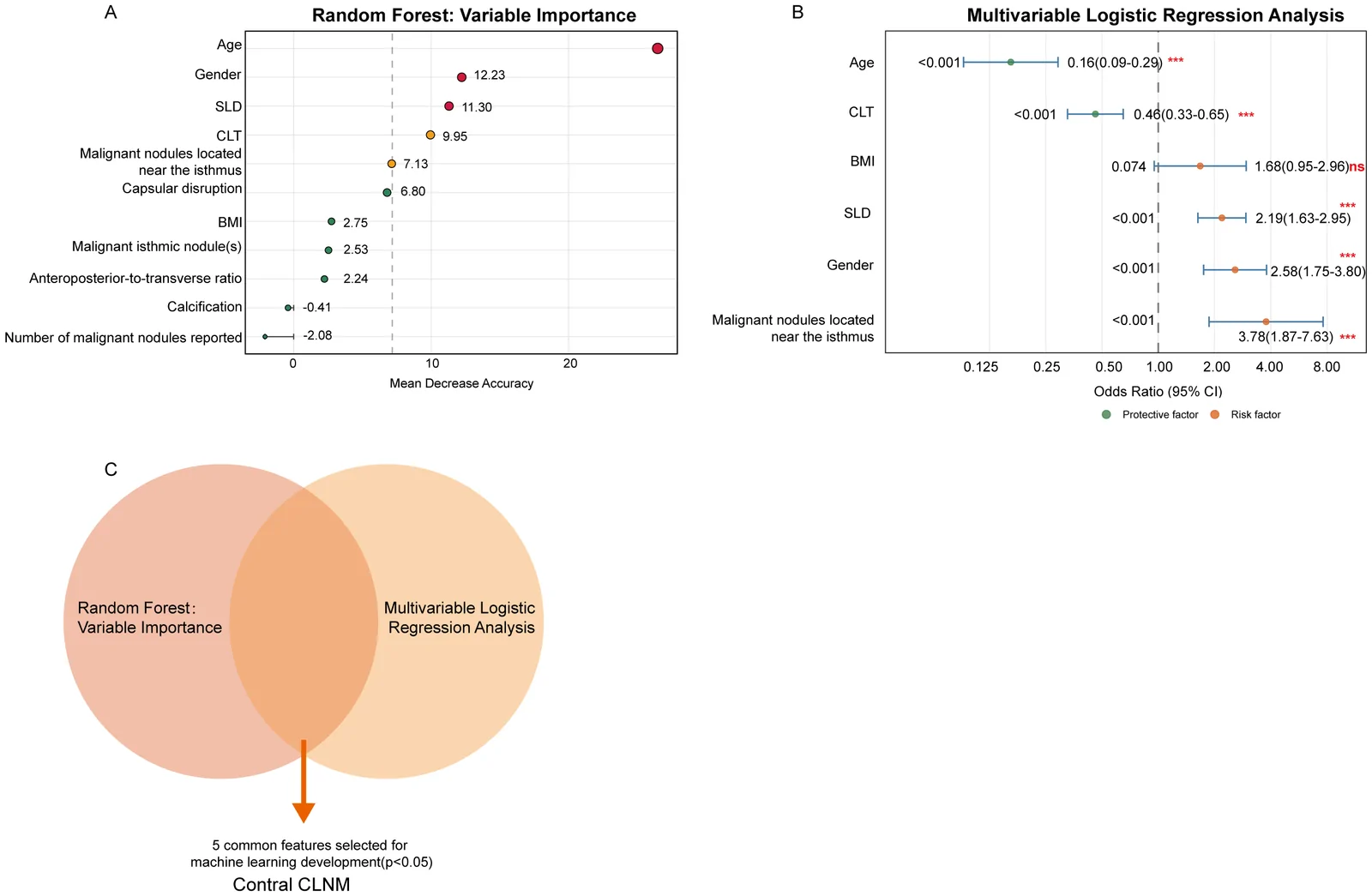
Comprehensive analysis of multiple variables to identify significant features associated with contralateral CLNM. **(A)** Random forest-based feature selection algorithm. Features marked in red and orange were identified as important features. **(B)** Forest plot of significant variables identified by multivariable regression analysis (P < 0.05). **(C)** Venn diagram illustrating the overlap of features selected by the random forest algorithm and multivariable logistic regression analysis.SLD, sum of the longest diameter; CLT, chronic lymphocytic thyroiditis; *p<0.05; **p<0.01; ***p<0.001; ns,not significant.

As illustrated in **Figures 2** and **3**, important variables were screened using two methods after multicollinearity assessment. Ultimately, four variables were incorporated into the prediction model for contralateral OTC, whereas five were included in the prediction model for contralateral CLNM.

**Figure 4:**
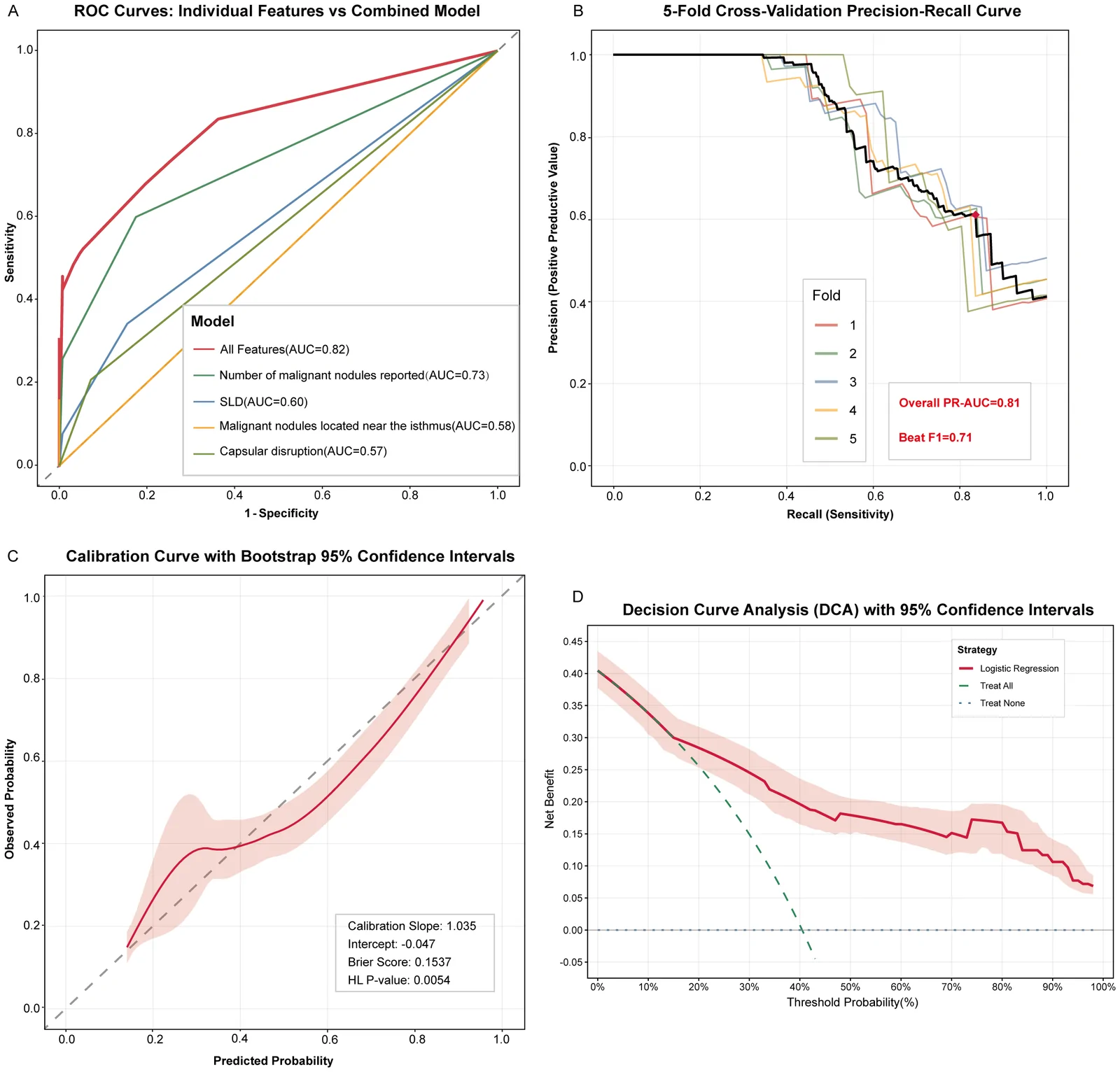
Model performance for predicting contarlateral OTC. **(A)** ROC curves of four feature combinations and individual features. **(B)** Precision-recall curve, **(C)** calibration curve, and **(D)** decision curve analysis (DCA) of the model, highlighting the excellent performance of the logistic regression model.SLD, sum of the longest diameter.

Prior to machine learning model development, receiver operating characteristic (ROC) curves were generated to compare the predictive performance of individual features and different feature combinations (**Figure 4A**). Subsequently, nine machine learning algorithms and a logistic regression model were trained and optimized using the entire dataset, with five-fold cross-validation applied for simultaneous hyperparameter tuning and initial performance estimation to maximize data utilization given the limited sample size. Stratified sampling based on outcome status ensured that the proportion of positive events in each fold remained consistent with the original cohort. The optimal algorithm was selected based on average performance across the five validation folds. Additionally, the area under the precision-recall curve (PR AUC) was calculated as a complementary performance metric.

To obtain a more reliable estimate of generalizable performance and correct for overfitting bias, the selected model underwent internal validation using 500 bootstrap resamples for optimism correction. Specifically, 500 bootstrap samples were generated with replacement from the original cohort, and the model was refitted in each resampled dataset. Performance metrics were calculated in both the bootstrap samples and the original cohort. The optimism-corrected AUC, along with its 95% confidence intervals derived from bootstrap distributions, was used as the primary discriminative performance metric.

Model calibration was assessed by plotting calibration curves based on bootstrap validation results, and the Hosmer–Lemeshow goodness-of-fit test was performed. Decision curve analysis (DCA) was conducted to evaluate the potential clinical net benefit of the models.

Finally, logistic regression-based prediction models were established to assess the individualized risks of contralateral OTC and contralateral CLNM in patients with T1b-T2 PTC. To facilitate clinical application, the prediction results were visualized as nomograms and web-based tools to assist clinicians in preoperative decision-making.

### 2.4 Statistical analysis

Continuous variables were first assessed for normality using the Shapiro-Wilk test. Normally distributed variables were presented as mean ± standard deviation(SD), while non-normally distributed variables were presented as median (interquartile range). Categorical variables were described using frequencies and percentages. Differences in categorical variables between two groups were compared using the chi-square test. For continuous variables, the two-tailed t-test was used for normally distributed data, and the Kruskal-Wallis rank sum test was used for non-normally distributed data. To exclude the influence of covariates during model construction, correlation analyses were performed as follows: Spearman’s correlation coefficient for continuous–continuous variable pairs, Cramer’s V for categorical–categorical variable pairs, and the point-biserial correlation coefficient for continuous–binary categorical variable pairs. Additionally, the variance inflation factor (VIF) was used to assess correlations among variables.

All statistical analyses were performed using R software version 4.5.3 (The R Foundation for Statistical Computing, Vienna, Austria; https://www.r-project.org/). A two-tailed P value < 0.05 was considered statistically significant.

## 3 Result

### 3.1 Data processing and features selection

As shown in **Figure 1**, a total of 932 patients were finally included in the development of the prediction model for contralateral OTC, while 730 patients were included in the development of the prediction model for contralateral CLNM. The remaining 202 patients were excluded from the CLNM model due to incomplete pathological data for contralateral central lymph nodes, mainly because they did not undergo bilateral central neck dissection. Baseline characteristics between included and excluded groups showed no significant differences in age, sex, tumor size, multifocality, or chronic lymphocytic thyroiditis (all P > 0.05), suggesting no substantial selection bias (**Supplementary Table 4**). (**Supplementary Table 4**)Clinicopathological characteristics and ultrasound imaging data were collected, and 11 candidate variables were initially included in the analysis(**Tables 1**, **2**). To detect and eliminate potential multicollinearity, Spearman correlation analysis and the point-biserial correlation analysis (threshold: |r| > 0.8),Cramer’s V analysis (threshold: |r| > 0.5) (**Figure 2A**) and VIF analysis (threshold: VIF > 10) were applied (**Supplementary Table 1**). No significant multicollinearity was observed among the 11 features.

**Table 2:** Patient demographics and baseline characteristics( contralateral CLNM).

Characteristics	No(n=367)	Yes(n=363)	P value
BMI			
Mean ± SD	23.46±3.13	23.67±3.62	0.083*
<24	213 (58.0%)	199 (54.8%)	
≥24 and<28	125 (34.1%)	117 (32.2%)	
≥28	29 (7.9%)	47 (12.9%)	
Age			<0.001
≤55	285 (77.7%)	343 (94.5%)	
>55	82 (22.3%)	20 (5.5%)	
Mean ± SD			
Gender			<0.001
Female	307 (83.7%)	226 (62.3%)	
male	60 (16.3%)	137 (37.7%)	
CLT			<0.001
No	188 (51.2%)	258 (71.1%)	
Yes	179 (48.8%)	105 (28.9%)	
Number of malignant nodules reported by ultrasound			0.138
1	237(64.6%)	227(62.5%)	
≥1	130(46.4%)	136 (47.5%)	
SLD (cm)			
Mean ± SD			<0.001*
Calcification reported by ultrasound			0.065
No	61 (16.6%)	43 (11.8%)	
Yes	306 (83.4%)	320 (88.2%)	
Anteroposterior-to-transverse ratio			0.023
<1	197 (53.7%)	225 (62.0%)	
≥1	170 (46.3%)	138 (38.0%)	
Malignant nodules located in the isthmus			0.1288
No	348 (94.8%)	333 (91.7%)	
Yes	19 (5.2%)	30 (8.3%)	
Malignant nodules located near the isthmus			<0.001
No	354 (96.5%)	323 (89.0%)	
Yes	13 (3.5%)	40 (11.0%)	
Capsular disruption			0.3891
No	61 (16.6%)	51 (14.0%)	
Yes	306 (83.4%)	312 (86.0%)	

CLNM, central lymph node metastasis; SD, standard deviation; CLT, chronic lymphocytic thyroiditis; SLD, sum of the longest diameter; *indicates the p values were obtained from the Kruskal–Wallis test, and others were obtained from the Chi-square tests.

Subsequently, the random forest algorithm was applied to calculate the importance score of each feature. Features were ranked according to their importance score, and those contributing to a cumulative importance of ≥ 80% were defined as core features, reflecting their major contribution to model prediction (**Figures 2B**, **3A**). Furthermore, multivariate logistic regression analysis was performed to identify statistically significant predictors (P<0.05), and forest plots were generated to illustrate the effect size of each selected feature on the study outcomes (**Figures 2C**, **3B**). Finally, Venn diagram was used to visualize the overlapping features identified by both the random forest algorithm and multivariate logistic regression. These shared features were retained for subsequent machine learning model construction (**Figures 2D**, **3C**).

### 3.2 Model construction and evaluation

**Figure 5:**
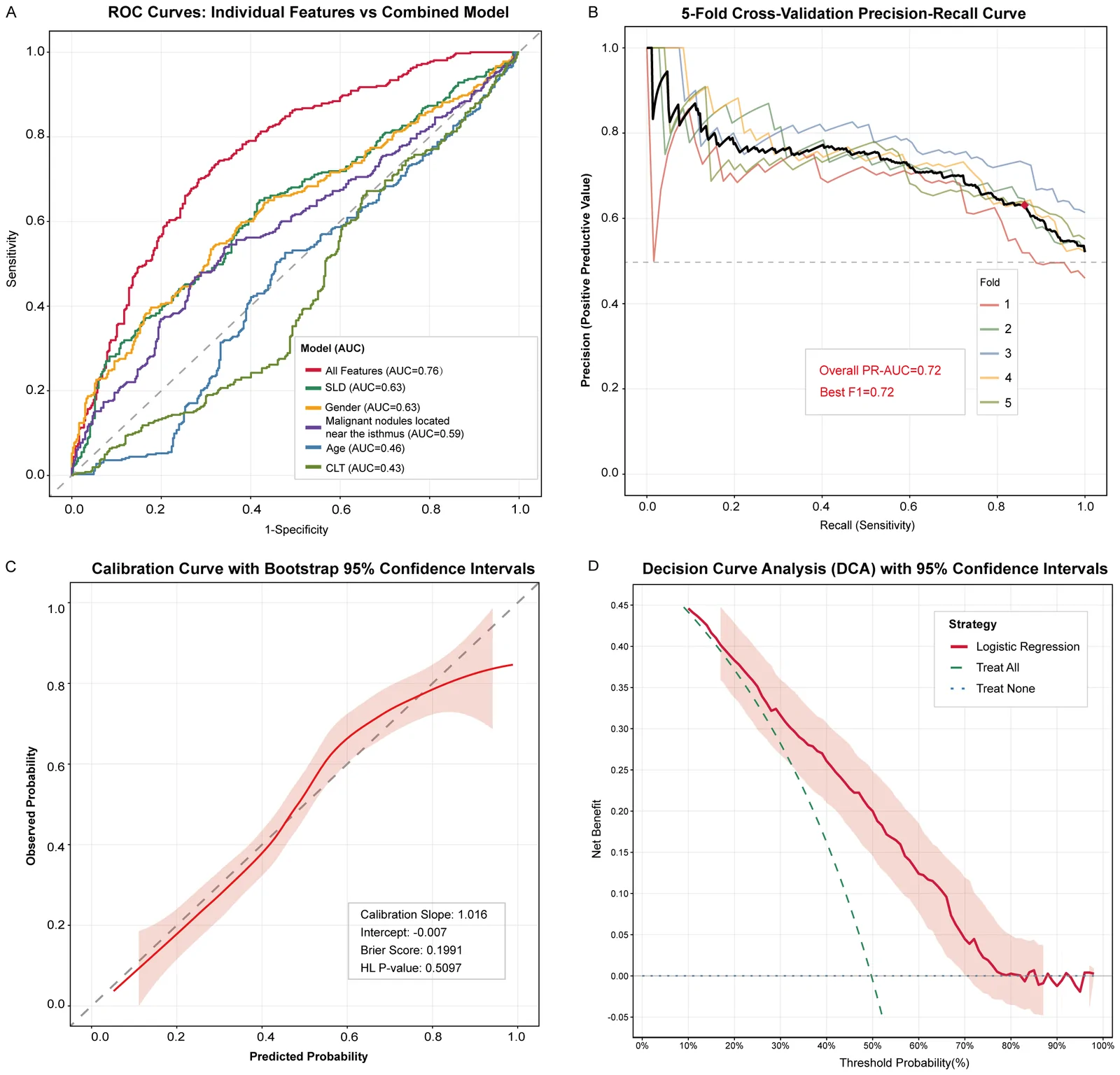
Model performance for predicting contralateral CLNM. **(A)** ROC curves of four feature combinations and individual features. **(B)** Precision-recall curve, **(C)** calibration curve, and **(D)** DCA of the model, highlighting the excellent performance of the logistic regression model. SLD, sum of the longest diameter; CLT, chronic lymphocytic thyroiditis.

Before implementing advanced machine learning techniques, we established a conventional logistic regression prediction model. Then the ROC curves of the combined feature model and individual features were compared. Our results demonstrated that the combined feature model exhibited superior predictive performance compared with any single feature alone (**Figures 4A**, **5A**).

Nine machine learning algorithms were employed to develop the prediction models, and model performance was evaluated using five-fold cross-validation. For the prediction of contralateral OTC, four features were included in the model development. The models achieved balanced accuracies ranging from 71.0% (95% CI: 67.8% to 74.2%) to 73.5% (95% CI: 71.6% to 75.3%), with AUC values ranging from 78.3% (95% CI: 71.5% to 85.1%) to 82.0% (95% CI: 81.5% to 82.6%) (**Supplementary Table 2**). Overall, these models showed favorable performance across multiple evaluation metrics, with logistic regression analysis showing outstanding performance across multiple indicators. For contralateral CLNM prediction, five features were included in model construction. The models achieved balanced accuracies ranging from 69.9% (95% CI: 68.1% to 73.4%) to 71.9% (95% CI: 69.1% to 75.4%), while the AUC values ranged from 71.9% (95% CI: 69.0% to 75.3%) to 79.1% (95% CI: 76.2% to 82.2%) (**Supplementary Table 3**). Among these models, glmBoost demonstrated the best overall performance.

The logistic regression model achieved an AUC of 79.0%, which was comparable to that of the glmBoost model (AUC of 79.1%). Considering its superior interpretability, ease of clinical application, and lower risk of overfitting, logistic regression was selected as the primary prediction tool in this study. Based on the selected variables, multivariate logistic regression models were established. The analysis revealed that malignant isthmic nodules, SLD, number of malignant nodules, and capsular disruption were positively associated with contralateral OTC (**Table 3**). In addition, isthmus-adjacent nodule, male sex, and SLD were positively associated with contralateral CLNM. Age > 55 years and CLT were negatively associated with contralateral CLNM (**Table 4**).

**Table 3:** Univariate and multivariable regression analysis for clinical factors associated with contralateral OTC.

	Univariate analysis		Multivariate analysis	
Characteristics	P value	95% CI	P value	95% CI
Malignant nodules located in the isthmus				
No		1.00 (Reference)		1.00 (Reference)
Yes	<0.001	8.015 (4.010-16.020)	<0.001	5.903 (2.869-12.149)
Capsular				
NO		1.00 (Reference)		1.00 (Reference)
Yes	0.005	1.686 (1.173-2.425)	0.02	1.577 (1.074-2.316)
SLD (cm)	<0.001	1.904 (1.532-2.368)	<0.001	1.811 (1.445-2.270)
Number of malignant nodules reported by ultrasound				
1		1.00 (Reference)		1.00 (Reference)
≥1	<0.001	2.582 (1.957-3.406)	<0.001	1.935 (1.445-2.270)

OTC, contralateral occult carcinoma; SLD, sum of the longest diameter.

**Table 4:** Univariate and multivariable regression analysis for clinical factors associated with contralateral CLNM.

	Univariate analysis		Multivariate analysis	
Characteristics	P value	95% CI	P value	95% CI
Gender				
Female		1.00 (Reference)		1.00 (Reference)
male	<0.001	3.102 (2.189-4.396)	<0.001	2.666 (1.815-3.916)
SLD (cm)				
Mean ± SD	<0.001	2.285 (1.755-2.977)	<0.001	2.274 (1.708-3.029)
Age				
≤55		1.00 (Reference)		1.00 (Reference)
>55	<0.001	0.203 (0.121-0.339)	<0.001	0.158 (0.089-0.282)
CLT				
No		1.00 (Reference)		1.00 (Reference)
Yes	<0.001	0.427 (0.315-0.580)	<0.001	0.474 (0.338-0.667)
Malignant nodules located near the isthmus				
No		1.00 (Reference)		1.00 (Reference)
Yes	<0.001	3.372 (1.772-6.419)	<0.001	4.117 (2.048-8.274)

CLNM, central lymph node metastasis; SLD, sum of the longest diameter;CLT, chronic lymphocytic thyroiditis.

### 3.3 Performance evaluation of the prediction model

The performance of the prediction models was assessed through discrimination and calibration. As described above, the AUC values for the prediction models for contralateral OTC and contralateral CLNM were 82.0% (95% CI:81.5% to 82.6%) and 79.0% (95% CI:76.0% to 81.9%), respectively, indicating satisfactory discriminatory capability. Furthermore, the diagnostic performance of the models was assessed using the area under the PR-AUC with five-fold cross-validation. A marked decline in precision was observed only at relatively high recall thresholds, indicating that the models maintained acceptable precision while detecting the majority of true positive cases (**Figures 4B**, **5B**). Model calibration was evaluated using 500 bootstrap resamples, which showed strong agreement between predicted probabilities and observed outcomes. The Hosmer-Lemeshow goodness-of-fit test yielded P values > 0.05, indicating no statistically significant difference between the predicted and actual probabilities. Collectively, these results confirmed the excellent calibration performance of the models (**Figures 4C**, **5C**).

**Figure 6:**
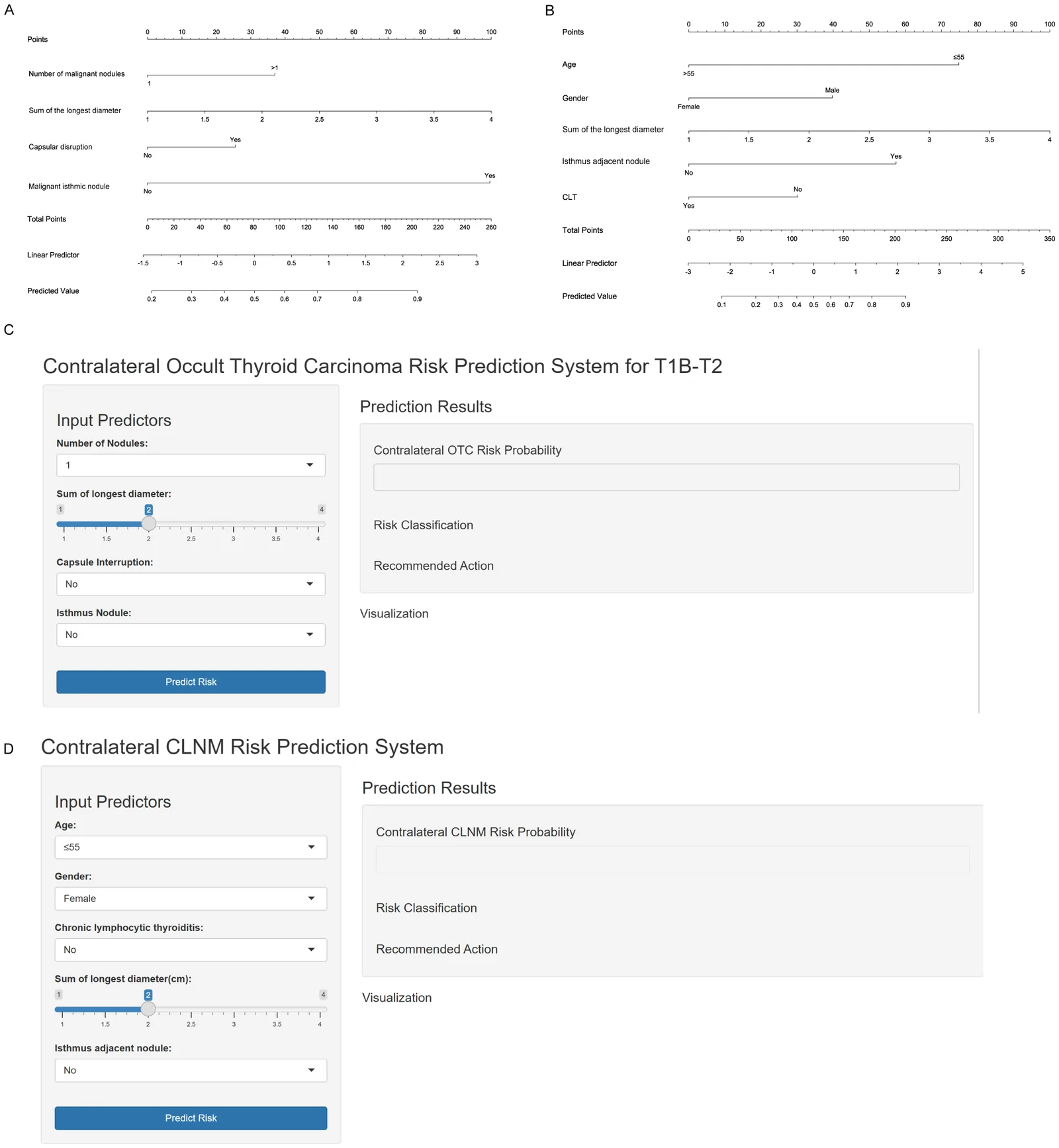
Nomograms and web-based applications for risk assessment of contralateral OTC and contralateral CLNM.**(A)** Nomogram for contralateral OTC prediction. **(B)** Nomogram for contralateral CLNM prediction. **(C)** Web application for contralateral OTC prediction. **(D)** Web application for contralateral CLNM prediction.

Finally, we evaluated the clinical utility of the nomograms using DCA. The results showed that the nomogram achieved the maximum net benefit across threshold probability ranges of 15%~100% for contralateral OTC and 0~80% for contralateral CLNM (**Figures 4D**, **5D**). This finding indicates that when the nomogram predicts a probability of contralateral thyroid carcinoma below 15%, model guided intervention does not confer a significant net benefit relative to the strategy of treating all patients. Conversely, when the predicted probability exceeds 15%, clinicians are encouraged to consider more intensive surveillance or proactive intervention, as the benefits of model based management outweigh those of alternative approaches. With respect to the prediction of contralateral lymph node metastasis, the nomogram exhibited a favorable net benefit across a broad threshold range from 0% to 80%. This suggests that even among patients with a very low estimated risk, the model retains the capacity to identify individuals who might derive benefit from prophylactic neck dissection, thereby supporting more nuanced and individualized surgical decision making. These indicate that the models possess favorable clinical applicability and may serve as effective tools for preoperative risk stratification and clinical decision support. To enhance the clinical implementation, we developed two nomograms and two web-based applications for individualized risk assessment (**Figure 6**). The online tools can be accessed at:1.contralateral OTC: https://liexiantuapp.shinyapps.io/contralateral-otc-T1bT2/; 2.contralateral CLNM: https://liexiantuapp.shinyapps.io/contralateral-clnm-T1bT2/.

## 4 Discussion

Over the past several decades, the incidence of thyroid cancer has increased steadily worldwide, with PTC accounting for more than 90% of all cases. This trend may be associated with the widespread application of ultrasound and changes in environmental factors. According to previous studies, the incidence of PTC has increased by 2.4-fold over recent decades, with the most prominent rise observed among patients with low-risk intrathyroidal T1-T2 tumors (23, 24). Despite the rising incidence, thyroid cancer-related mortality remains stable, with a 10-year survival rate exceeding 90%.

Surgery remains the only curative treatment for thyroid cancer. However, the optimal extent of surgical intervention continues to be controversial due to the tricky balance between disease recurrence and postoperative complications (25). In the 2009 ATA guidelines, total thyroidectomy was recommended for patients with PTC tumors larger than 1 cm in diameter. However, the 2016 ATA guidelines revised this recommendation, suggesting that thyroid lobectomy alone may be sufficient for low-risk patients with tumors measuring 1~4 cm (25, 26). Evidence indicates that total thyroidectomy is associated with a higher incidence and greater severity of surgical complications compared with lobectomy (27). In recent years, various refined surgical techniques, such as energy-based devices and meticulous capsular dissection, have been introduced to minimize intraoperative trauma and improve hemostasis, potentially mitigating some of these morbidity risks (28).On the other hand, proponents of total thyroidectomy argue that lobectomy may increase recurrence risk in patients with PTC >1 cm (29). The most recent 2025 ATA guidelines suggested that total thyroidectomy should be considered in patients with suspicious contralateral nodules to facilitate potential radioactive iodine (RAI) therapy and improve long-term surveillance (8). These considerations underscore the importance of individualized management based on tumor stage and patient-specific risk profiles.

Contralateral OTC and lymph node metastasis are important contributors to postoperative recurrence and the need for second surgery in patients with thyroid cancer (28, 30, 31). Previous studies have reported that the incidence of contralateral OTC can reach 20~40%. This indicates that, if all patients undergo prophylactic total thyroidectomy, approximately 60~80% may be overtreated. Therefore, developing an accurate risk prediction model for contralateral OTC has important clinical significance for guiding surgical extent decisions in these patients (32, 33).

In this study, approximately 41.31% (385) were found to have OTC on postoperative pathological examination. Using the random forest algorithm and multivariate logistic regression analysis, four predictive features were ultimately identified for model construction. The final logistic regression model achieved an AUC of 82.0%, and bootstrap resampling demonstrated satisfactory calibration.

Additionally, a separate prediction model was developed for contralateral CLNM. A total of 730 patients were enrolled in this analysis, with approximately 49.73% (363) were confirmed to have contralateral CLNM. After feature selection, five variables were incorporated into the final model. The glmBoost model and the logistic regression model achieved comparable AUC (79.1% and 79.0%, respectively), with no statistically significant difference. Considering the superior interpretability, better clinical applicability, and lower risk of overfitting, logistic regression was selected as the final prediction tool.

Our study demonstrated that malignant isthmic nodule, SLD, number of malignant nodules, and capsular disruption were positively associated with contralateral OTC, which is consistent with previous studies (10, 12, 13). Chen et al. reported that primary tumor multifocality, the presence of benign nodules in the contralateral lobe, and larger nodule size were positively associated with contralateral OTC (12). In addition to previously reported factors, our study further identified malignant isthmic nodule as a potential independent predictor of contralateral OTC (34).

In this study, the model established for CLNM prediction revealed that isthmus-adjacent malignant nodules, male sex, and SLD were positively associated with contralateral CLNM, whereas age >55 years and CLT were negatively associated with contralateral CLNM. These findings are consistent with previous studies (16, 19, 35). Notably, the association between CLT and CLNM remains controversial. Some studies suggested that CLT may exert a protective effect against CLNM (36, 37), possibly through immune-mediated mechanisms that suppress tumor transformation and progression (38). In contrast, other studies have reported that patients with CLT are more likely to be diagnosed with PTC (39). In this study, we found that CLT was not significantly associated with contralateral OTC, but negatively associated with contralateral CLNM, which is in line with several previous studies (37, 39).

Compared with previously published prediction models that focused on a single outcome (12, 16), all features included in this study were derived from routinely available preoperative clinical and ultrasound data, thereby enhancing the practicality and accessibility of the models in real-world clinical settings. Currently, prediction models specifically developed for patients with T1b to T2 stage disease remain lacking. Therefore, our study specifically focused on this patient population, for whom individualized treatment remains an unmet clinical need.

This study employed a dual feature selection approach combining random forest analysis and logistic regression, which enhanced the robustness of feature selection. Finally, the stability of the model was assessed using calibration curves. The DCA demonstrated favorable clinical utility of the models.

In summary, our findings suggest that the proposed models provide clinicians with a simple and feasible preoperative assessment tool for identifying patients at high risk of contralateral OTC and contralateral CLNM. These tools may help optimize surgical decision-making and reduce unnecessary surgical risks in patients with T1b-T2 stage disease.

Nevertheless, several limitations should be acknowledged. First, selection bias is inherent to this single-center retrospective design. Second, the inclusion of only T1b-T2 stage patients limits generalizability to other PTC subgroups; thus, the nomograms are best suited for patients with suspected contralateral lesions or uncertain surgical plans, rather than low-risk lobectomy candidates. Third, excluding patients with incomplete records may have yielded a relatively homogeneous cohort from a tertiary center, potentially underrepresenting community hospital heterogeneity. Additionally, as the total thyroidectomy cohort may have a higher incidence of contralateral lesions than the general surgical population, the model predictions could be overestimated. Clinicians should interpret results in light of individual patient factors. Ultimately, this nomogram is intended to assist, not replace, surgical judgment. To address these limitations, we are planning to conduct multicenter prospective studies to further validate the accuracy and generalizability of our models in broader clinical settings.

## 5 Conclusion

This study established a preoperative prediction model for assessing the risks of contralateral OTC and contralateral CLNM in patients with unilateral T1b-T2 stage PTC. The models may assist clinicians in formulating individualized surgical strategies and promoting the implementation of precision medicine in thyroid cancer management.

## Data Availability

The raw data supporting the conclusions of this article will be made available by the authors, without undue reservation.
